# Sodium Butyrate More Effectively Mitigates the Negative Effects of High-Concentrate Diet in Dairy Cows than Sodium *β*-Hydroxybutyrate via Reducing Free Bacterial Cell Wall Components in Rumen Fluid and Plasma

**DOI:** 10.3390/toxins13050352

**Published:** 2021-05-14

**Authors:** Yongjiang Wu, Yawang Sun, Ruiming Zhang, Tianle He, Guohao Huang, Ke Tian, Junhui Liu, Juncai Chen, Guozhong Dong

**Affiliations:** 1 College of Animal Science and Technology, Southwest University, Chongqing 400715, China; wuyongjiang@email.swu.edu.cn (Y.W.); syaw507@swu.edu.cn (Y.S.); capprice@email.swu.edu.cn (R.Z.); h1971763109@email.swu.edu.cn (T.H.); hgh328001820@email.swu.edu.cn (G.H.); a6101004@edu.gifu-u.ac.jp (K.T.); junhui.liu@cragenomica.es (J.L.); juncaichen@swu.edu.cn (J.C.); 2 United Graduate School of Agricultural Science, Gifu University, Gifu 501-1193, Japan; 3 Centre for Research in Agricultural Genomics (CRAG), University Autonomous of Barcelona, 08193 Barcelona, Spain

**Keywords:** sodium butyrate, sodium *β*-hydroxybutyrate, lipopolysaccharide, peptidoglycan, lipoteichoic acid, dairy cows

## Abstract

The present study was aimed at investigating the effects of sodium butyrate and sodium *β*-hydroxybutyrate on lactation and health of dairy cows fed a high-concentrate (HC) diet. Eighty mid-lactation dairy cows with an average milk yield of 33.75 ± 5.22 kg/d were randomly allocated to four groups (*n* = 20 per group) and were fed either a low-concentrate (LC) diet, a HC diet, the HC diet with 1% sodium butyrate (HCSB), or the HC diet with 1% sodium *β*-hydroxybutyrate (HCHB). The feeding trial lasted for 7 weeks, with a 2-week adaptation period and a 5-week measurement period, and the trial started from 96 ± 13 d in milk. Sodium butyrate supplementation delayed the decline in milk production and improved milk synthesis efficiency and milk fat content. Additionally, it decreased the proinflammatory cytokines and acute phase proteins (APPs) in plasma, the leucocytes in blood, the somatic cell count (SCC) in milk, and the gene expression of pattern recognition receptors (PRRs) and proinflammatory cytokines in the mammary gland, due to decreasing the contents of bacterial cell wall components (lipopolysaccharide, LPS; peptidoglycan, PGN; and lipoteichoic acid, LTA) in the rumen and plasma, compared with the HC diet. Sodium *β*-hydroxybutyrate supplementation also improved milk yield, milk synthesis efficiency and milk fat content and partially reduced the adverse effects caused by the HC diet, but it had no effect on decreasing bacterial cell wall components in the rumen and plasma, compared with the HC diet. Collectively, both sodium butyrate and sodium *β*-hydroxybutyrate mitigated the negative effects of HC diet on lactation and health of dairy cows, with sodium butyrate being more effective than sodium *β*-hydroxybutyrate.

## 1. Introduction

To increase milk production and maximize economic benefits, dairy cows are usually fed a high-concentrate (HC) diet. However, HC diet often results in subacute ruminal acidosis (SARA). During the occurrence and development of SARA, the microbial communities in the rumen are disturbed, and the richness and diversity of rumen microbes are decreased [1,2]. Meanwhile, large amounts of bacterial cell wall components are released into rumen fluid [3]. Bacterial cell wall components mainly include lipopolysaccharide (LPS), the endotoxin of Gram-negative bacteria (GNB) [3]; lipoteichoic acid (LTA), the endotoxin of Gram-positive bacteria (GPB) [3]; and peptidoglycan (PGN), the structural polysaccharide from both GNB and GPB [4,5]. LPS, LTA and PGN are pathogen-associated molecular patterns (PAMPs) [5,6], and thus they can activate corresponding pattern recognition receptors (PRRs) [7,8,9]. LPS, LTA and PGN predominantly activate Toll-like receptor (TLR) 4 [7], TLR2 [8] and nucleotide-binding oligomerization domain (NOD) 1 [9], respectively, resulting in inflammatory responses. SARA also increases the permeability of rumen epithelium by impairing rumen epithelial barrier function [10]. As a result, significant amounts of bacterial cell wall components translocate into blood circulation, inducing systemic and local inflammations [11]. Up to now, LPS has received a lot of attention due to its strong proinflammation activity. Numerous studies [12,13] have demonstrated that HC diet feeding significantly increases the concentrations of LPS in the rumen and plasma of ruminants and induces inflammation in the rumen epithelium, the mammary gland, the liver, and so on. Besides, several studies [14,15] have also suggested that HC diet feeding significantly increases the concentration of D-glutamyl-*meso*-diaminopimelic acid (iE-DAP), a core component of PGN, in the rumen and plasma of dairy cows and goats and elicited inflammation in the mammary gland and liver. Whether HC diet increases the concentrations of PGN and LTA in the rumen and plasma of dairy cows has yet to be established.

Sodium butyrate, as a histone deacetylase (HDAC) inhibitor, facilitates gene transcription by inhibiting HDAC [10,16] and possesses anti-inflammatory, anti-oxidative, anti-apoptotic and other activities [12]. In addition, oral administration of sodium butyrate promotes ruminal development and improves production performance and health status in young calves [10,17]. Sodium butyrate enhances the expression of lactation-related genes in bovine mammary epithelial cells in vitro [18,19]. Supplementation of sodium butyrate into HC diet increases milk yield and milk fat content in dairy cows [20]. Furthermore, sodium butyrate attenuates the adverse effects including mastitis, hepatitis, rumen epithelial impairment, and so on, caused by HC diet in ruminants, since sodium butyrate increases rumen pH and decreases rumen epithelial permeability and the concentrations of LPS and iE-DAP in the rumen and plasma [10,14,17]. However, the effects of sodium butyrate addition into HC diet on the concentrations of PGN and LTA in the rumen and plasma remain unclear.

After butyrate is absorbed by ruminal epithelium, it is rapidly metabolized to *β*-hydroxybutyrate [21] which in turn increases the concentration of *β*-hydroxybutyrate in the blood [20]. *β*-Hydroxybutyrate accounts for 75% of blood ketone bodies [22]. Therefore, its concentration in blood is a measure of blood ketone body levels. In general, *β*-hydroxybutyrate concentration over 1.2 mmol/L in blood is used as a benchmark for ketosis in dairy cows [23]. Similar to sodium butyrate, *β*-hydroxybutyrate is also a HDAC inhibitor [24,25] involved in the regulation of gene transcription. Additionally, *β*-hydroxybutyrate is an essential carrier of energy from the liver to peripheral tissues and a precursor substance of milk fat synthesis [25,26] and thus can enhance milk fat synthesis in bovine mammary epithelial cells [27,28]. Furthermore, *β*-hydroxybutyrate can attenuate inflammatory disease via blocking NOD-like receptor (NLR) family pyrin domain containing 3 (NLRP3) inflammasome and activating G protein-coupled receptor (GPR) 109A [29,30]. Therefore, we speculated that *β*-hydroxybutyrate might exhibit stronger activities of anti-inflammation and pro-lactation than sodium butyrate.

The present study was aimed at investigating the effects of HC diet on the concentrations of bacterial cell wall components (LPS, PGN, and LTA) in the rumen and plasma as well as the associated adverse effects on the health and lactation of dairy cows. Meanwhile, sodium butyrate and sodium *β*-hydroxybutyrate were added into HC diet to compare their inhibitory effects on the adverse effects caused by HC diet.

## 2. Results

### 2.1. Production Performance and Milk Components

**Figure 1:**
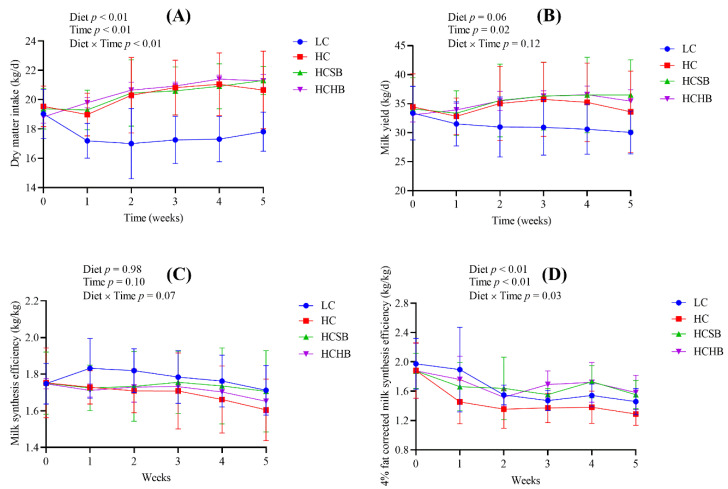
The dry matter intake (DMI, **A**), milk yield (**B**), milk synthesis efficiency (**C**), and 4% fat corrected milk (FCM) synthesis efficiency (**D**) of dairy cows. Milk synthesis efficiency and 4% FCM synthesis efficiency were calculated as milk yield and 4% FCM yield divided by DMI, respectively. LC: low concentrate diet; HC: high concentrate diet; HCSB: HC supplemented with 1% sodium butyrate; HCHB: HC supplemented with 1% sodium *β*-hydroxybutyrate. The data are analyzed using the general linear model with repeated measures and presented as means ± standard deviation. A statistically significant difference is presented as a probability value less than 0.05 (*p* < 0.05, n = 20).

Diet, time and diet × time interaction significantly affected the dry matter intake (DMI) (Figure 1A). There were no significant differences in the DMI among the HC group, HC with sodium butyrate (HCSB) group, and HC with sodium *β*-hydroxybutyrate (HCHB) group. The DMI of these three groups was significantly higher than that of the low-concentrate (LC) group. Diet tended to affect milk yield (Figure 1B). The milk yield of the LC group exhibited a steep drop for the first and second week before decreasing slowly. The milk yield of the HC group peaked at the third week and began a gradual decline. The milk yield of the HCSB group also reached a peak at the third week and remained at the peak level until the end of the trial. The milk yield of the HCHB group gradually reached a peak at the fourth week and then began to decline. Time significantly affected milk yield, but diet × time interaction did not affect milk yield (Figure 1B). Diet, time and diet × time interaction did not affect milk synthesis efficiency (Figure 1C), but significantly affected 4% fat corrected milk (FCM) synthesis efficiency (Figure 1D). The milk synthesis efficiency and 4% FCM synthesis efficiency in the HC group were the lowest in the four groups (Figure 1C,D).

**Figure 2:**
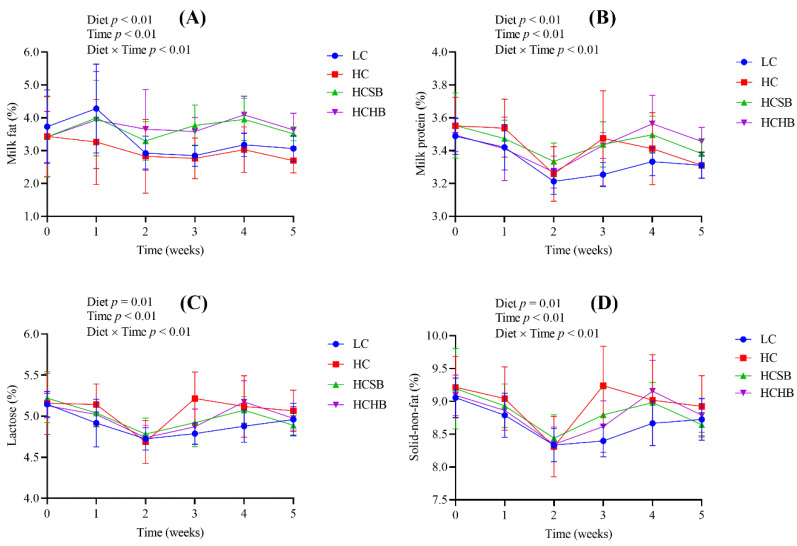
The contents of milk fat (**A**), milk protein (**B**), lactose (**C**), and solid-non-fat (**D**) in milk. LC: low concentrate diet; HC: high concentrate diet; HCSB: HC supplemented with 1% sodium butyrate; HCHB: HC supplemented with 1% sodium *β*-hydroxybutyrate. The data are analyzed using the general linear model with repeated measures and presented as means ± standard deviation. A statistically significant difference is presented as a probability value less than 0.05 (*p* < 0.05, n = 20).

Diet, time and diet × time interaction significantly affected milk components including milk fat, milk protein, lactose, and solid-non-fat (Figure 2). The milk fat contents in the HCSB and HCHB groups were significantly higher than those in the LC and HC groups. All the four milk components were not significantly different between the HCSB and HCHB groups.

### 2.2. Rumen pH and the Concentrations of LPS, PGN and LTA in Rumen Fluid and Plasma

As shown in Table 1, the rumen pH of the HC group was lower than that of LC group. Compared with the HC group, the pH of rumen fluid in the HCSB and HCHB groups significantly increased. The pH of rumen fluid in the HCSB group was significantly higher than that in the HCHB group. Compared with the LC group, all the concentrations of LPS, PGN and LTA in rumen fluid and plasma significantly increased in the HC, HCSB and HCHB groups, except the PGN in the plasma in the HCSB group. Compared with the HC group, all the concentrations of LPS, PGN and LTA in rumen fluid and plasma significantly decreased in the HCSB group, except the PGN in the rumen fluid, and all the concentrations of LPS, PGN and LTA in rumen fluid and plasma were not significantly different in the HCHB group. All the concentrations of LPS, PGN and LTA in rumen fluid and plasma in the HCSB group were lower than those in the HCHB group, except the PGN in rumen fluid.

### 2.3. Concentrations of Proinflammatory Cytokines and Acute Phase Proteins (APPs) in Plasma

As shown in Table 2, compared with the LC group, the concentrations of the four proinflammatory cytokines including interleukin (IL)-1*β*, IL-6, IL-8, and tumor necrosis factor (TNF)-*α* significantly increased in the HC group, and IL-1*β* in the HCSB group and IL-1*β* and IL-8 in the HCHB group significantly increased. Compared with the HC group, the concentrations of the four proinflammatory cytokines in the HCSB group significantly decreased, and IL-6 in the HCHB group significantly decreased. The concentration of IL-1*β* in the HCSB group was significantly lower than that in the HCHB group. Compared with the LC group, the concentrations of the APPs including LPS binding protein (LBP), serum amyloid A (SAA), C-reactive protein (CRP), and haptoglobin (Hp) significantly increased in the HC group, and LBP in the HCSB group and LBP, SAA and CRP in the HCHB group significantly increased. Compared with the HC group, the concentrations of CRP and Hp in the HCSB group and Hp in the HCHB group significantly decreased. All the four APPs were not significantly different between the HCSB and HCHB groups.

### 2.4. Blood Leucocyte Indexes and Milk Somatic Cell Count (SCC)

**Figure 3:**
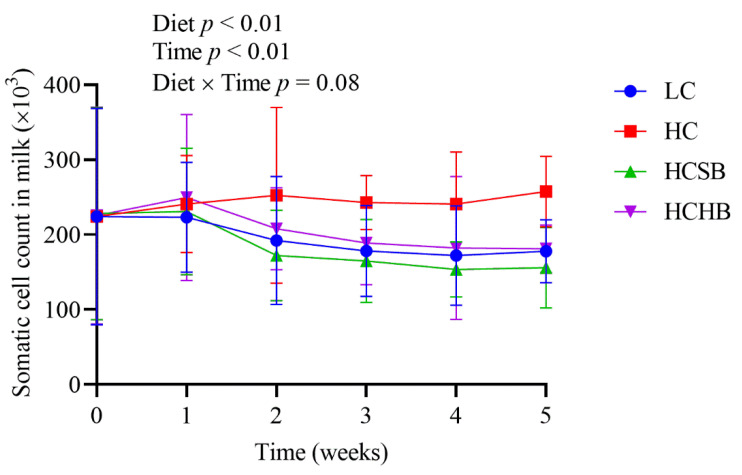
The somatic cell count in milk. LC: low concentrate diet; HC: high concentrate diet; HCSB: HC supplemented with 1% sodium butyrate; HCHB: HC supplemented with 1% sodium *β*-hydroxybutyrate. The data are analyzed using the general linear model with repeated measures and presented as means ± standard deviation. A statistically significant difference is presented as a probability value less than 0.05 (*p* < 0.05, n = 10).

As shown in Table 3, compared with the LC group, all the leucocytes (lymphocyte, monocyte, and neutrophil) significantly increased in the HC group. Compared with the HC group, both the number of white blood cells, lymphocytes, and monocytes in the HCSB group and the number of white blood cells and lymphocytes in the HCHB group significantly decreased. All the leucocytes were not significantly different between the HCSB and HCHB groups. Both diet and time significantly affected SCC in milk, but the diet × time interaction did not affect SCC (Figure 3). The SCC in the HC group was significantly higher than that in the other three groups from the second week.

### 2.5. Blood Glucose and Blood Ketone Bodies

As shown in Table 4, the blood glucose concentrations among the groups were not significantly different. Compared with the LC group, the blood ketone bodies of the HC group significantly decreased, the blood ketone bodies of the HCSB group did not significantly change, and the blood ketone bodies of the HCHB group significantly increased. Compared with the HC group, the blood ketone bodies significantly increased in both the HCSB and HCHB groups. The blood ketone bodies of the HCHB group were significantly higher than those of the HCSB group.

### 2.6. Gene Expression of PRRs and Proinflammatory Cytokines in Milk Somatic Cells

**Figure 4:**
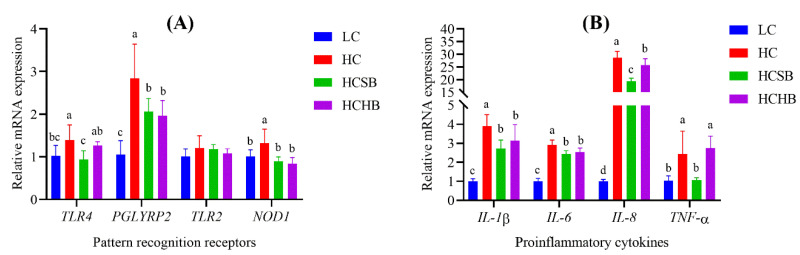
The gene expression of pattern recognition receptors (**A**) and proinflammatory cytokines (**B**) in milk somatic cells. LC: low concentrate diet; HC: high concentrate diet; HCSB: HC supplemented with 1% sodium butyrate; HCHB: HC supplemented with 1% sodium *β*-hydroxybutyrate; *TLR4*: Toll-like receptor 4; *PGLYRP2*: Peptidoglycan recognition protein 2; *TLR2*: Toll-like receptor 2; *NOD1*: nucleotide binding oligomerization domain containing 1; *IL-1β*: interleukin-1*β*; *IL-6*: interleukin-6; *IL-8*: interleukin-8; *TNF-α*: tumor necrosis factor-*α*. The data are presented as means ± standard deviation. For each gene, the columns marked without a common lowercase letter (a, b, and c) on the top indicate significant difference (*p* < 0.05, n = 6), based on one-way analysis of variance followed by Duncan’s multiple comparison test.

Compared with the LC group, the gene expression of PRRs including *TLR4*, *NOD1* and peptidoglycan recognition protein 2 (*PGLYRP2*) significantly increased in the HC group, and the gene expression of *PGLYRP2* also significantly increased in the HCSB and HCHB groups (Figure 4A). Compared with the HC group, the gene expression of the three PRRs (*TLR4*, *PGLYRP2* and *NOD1*) significantly decreased in the HCSB group, and the gene expression of *PGLYRP2* and *NOD1* also significantly decreased in the HCHB group (Figure 4A). The gene expression of *TLR4* in the HCSB group was significantly lower than that in the HCHB group. Compared with the LC group, the gene expression of all proinflammatory cytokines (*IL-1β*, *IL-6*, *IL-8*, and *TNF-α*) significantly increased in the HC, HCSB and HCHB groups (Figure 4B), except for the *TNF-α* in the HCSB group. Compared with the HC group, the gene expression of all proinflammatory cytokines significantly decreased in the HCSB and HCHB groups (Figure 4B), except for the *TNF-α* in the HCHB group. The gene expression of *IL-8* and *TNF-α* in the HCSB group was significantly lower than that in the HCHB group.

## 3. Discussion

In dairy production, dairy cows are commonly fed HC diet to meet the needs of energy and nutrients during lactation to increase milk yield. In this study, the milk yield in the HC group was higher than that in the LC group, which was consistent with the results of previous studies [31,32]. Although HC diet increased milk yield of dairy cows, it resulted in adverse effects on milk components. Numerous studies [33,34] have indicated that HC diet decreased milk fat content. In this study, the milk fat content in the HC group tended to be lower than that in the LC group throughout the experimental period, which agrees with previous studies [33,34]. Dairy cows fed LC diet often have an energy deficit in the lactation period, therefore, fat in adipose tissue is mobilized to supply energy. In this case, large amounts of ketone bodies are generated during the process of fat mobilization for energy supply [35]. Thus, the ketone body concentrations in the blood of the LC group were higher than those of the HC group in the present study, which confirmed previous findings [36].

HC diet is rapidly fermented by rumen microbes, generating large amounts of volatile fatty acids and resulting in a drop in ruminal pH. The low pH environment of rumen usually causes massive deaths of rumen microbes intolerant to low pH and great proliferation of rumen microbes tolerant to low pH. Large amounts of bacterial cell wall components are released into the rumen during the process of death and/or proliferation of rumen microbes [3]. HC diet resulted in increased rumen LPS concentration [37]. In our study, the HC diet significantly decreased rumen pH and increased rumen LPS concentration, which was consistent with numerous reports [38,39]. Besides, we further measured the concentrations of LTA and PGN in the rumen and plasma when the dairy cows were fed HC diet and found that the concentrations of both LTA and PGN significantly increased. Several studies have shown that HC diet led to an increase of rumen iE-DAP (the core component of PGN) concentration in dairy goats and cows [40,41]. Therefore, taken together, the present and previous study findings indicated that HC diet caused a comprehensive increase of bacterial cell wall components including LPS, LTA and PGN in the rumen.

LPS, PGN and LTA are PAMPs [5,6]. All of them can act as antigens and will induce an inflammatory response if present in the peripheral blood, resulting in injury to the body cells and tissues [5,6]. High concentration of LPS induced by HC diet usually results in ruminal epithelium injury, such as inflammation [42], apoptosis, high osmolarity [43], etc. Compromised rumen epithelium barrier allows the PAMPs to easily cross the rumen epithelium into the peripheral blood [3]. Thus, HC diet not only elevates LPS concentration in rumen fluid, but also results in a rise in plasma [1,2]. Several studies have suggested that HC diet also led to an increase of iE-DAP in the rumen and plasma [14,40]. In this present study, the HC diet significantly increased LPS concentration in plasma, which was in line with previous reports [1]. Our study also showed the HC diet significantly increased concentrations of LTA and PGN in plasma. Elevated PAMP concentrations in blood circulation can cause systemic and local immune responses [44,45] and significantly increase the concentrations of proinflammatory cytokines (IL-1*β*, IL-6, IL-8, and TNF-*α*) and APPs (LBP, SAA, CRP, and Hp) [1,2,3]. In the present study, the HC diet significantly increased the concentrations of proinflammatory cytokines and APPs in plasma, which was consistent with previous studies [1,2,3]. In addition, when immune responses are activated, the number of immune cells will markedly increase in the blood. Thus, the immune cell populations are usually used to predict the infectious condition of the animal body. The present study showed that the HC diet significantly increased the amounts of immune cells in the blood, which further demonstrated that the increase of PAMPs caused by the HC diet resulted in systemic inflammation in dairy cows. When the PAMPs are transported into the udder through blood circulation, they can induce inflammatory responses of mammary gland cells by activation of corresponding PRRs [8,9,46]. Both TLR4 and TLR2 are PRRs, which mainly are activated by LPS and LTA [8,47], respectively. NOD1 and PGLYRP2 are also PRRs and are responding to PGN stimulation [9,48]. After being activated by PAMPs, PRRs can promote the expression and release of proinflammatory cytokines in mammary gland cells. The expression pattern of milk protein genes, epithelial cell maker genes and RNA transcriptome was similar between milk somatic cells and the mammary tissue [49,50]. Thus, milk somatic cells can be used as effective and alternative samples to study the gene expression profiling of the mammary gland tissue without the need to conduct a tissue biopsy [49,50,51]. In this study, the HC diet significantly increased the expression of PRR genes including *TLR4*, *PGLYRP2* and *NOD1* and the expression of proinflammatory cytokine genes including *IL-1β*, *IL-6*, *IL-8* and *TNF-α* in milk somatic cells, indicating that the HC diet could induce inflammation through increasing PAMPs (LPS, LTA, and PGN) in the rumen and plasma. The increased proinflammatory cytokines recruit numerous immune cells to inflamed tissues [52]. Thus, numerous immune cells accumulated in the udder and then resulted in increased SCC in milk in the present study.

Butyrate, a short chain fatty acid produced during rumen fermentation is mainly used for energy supply in ruminants. In this study, the milk yield of the HC group began to decline at the third week, but the milk yield remained constant at a high level when sodium butyrate was added in the HC diet. Sodium butyrate also increased milk production efficiency and milk fat content in the present study. Sodium butyrate has the ability to increase milk fat synthesis of bovine mammary epithelial cells in vitro through activation of GPR41 and its downstream signaling pathways [19]. Sodium butyrate added in either HC diet or LC diet of dairy cows tended to increase the synthesis efficiency of 4% FCM and significantly increased milk fat content [20], which was in agreement with our results. Dietary addition of sodium butyrate increased *β*-hydroxybutyrate concentration in the peripheral blood in dairy cows [20], corroborating the results of our present study. HC diet supplemented with sodium *β*-hydroxybutyrate increased *β*-hydroxybutyrate concentration in the blood in the present study. Furthermore, the ketone bodies generated for the HC diet supplemented with sodium butyrate were less than that supplemented with sodium *β*-hydroxybutyrate in this study. Although both sodium butyrate and sodium *β*-hydroxybutyrate increased *β*-hydroxybutyrate concentrations in the blood, the values were below 1.2 mmol/L, the benchmark for ketosis. *β*-Hydroxybutyrate as the precursor substance of milk fat synthesis can promote the synthesis and secretion of milk fat [28,53]. Moreover, sodium *β*-hydroxybutyrate tended to increase milk yield and milk production efficiency, but the effects were not greater than those of sodium butyrate. Taken together, these results suggest that sodium butyrate is more effective than sodium *β*-hydroxybutyrate in improving lactation, and sodium butyrate supplementation results in less blood ketone bodies than sodium *β*-hydroxybutyrate supplementation.

Sodium butyrate and sodium *β*-hydroxybutyrate not only promoted lactation, but also attenuated inflammation in dairy cows in our study. Sodium butyrate could inhibit the inflammation induced by iE-DAP and LPS in bovine hepatocytes via suppressing NOD1and NOD1-mediated inflammatory molecules (IL6, IL8, TNF-α, etc.) expression in vitro [12,16]. Similarly, several studies have suggested that supplementation of sodium butyrate could comprehensively reduce the body injuries induced by HC diet in ruminants. These injuries included inflammation in the mammary gland [12,14], liver [15], rumen epithelium [17]; apoptosis in mammary cells [54] and hepatocytes [13]; and impairment of ruminal epithelium barrier function [10]. *β*-Hydroxybutyrate also possesses an anti-inflammatory activity via activating GPR109A [30] and decreasing the expression of innate immune genes [55]. In the present study, we further concluded that both sodium butyrate and sodium *β*-hydroxybutyrate attenuated the inflammation induced by HC diet, with sodium butyrate being more effective than sodium *β*-hydroxybutyrate. There are several reasons. Firstly, sodium butyrate improved the rumen pH and comprehensively decreased the bacterial cell wall components (LPS, PGN and LTA) in rumen fluid and plasma, which was consistent with previous studies [10,14,15]. Sodium *β*-hydroxybutyrate also improved rumen pH, but to a less extent than sodium butyrate. Furthermore, sodium *β*-hydroxybutyrate had not significant effects on all the three bacterial cell wall components in rumen fluid. Secondly, consistent with previous studies [12,17,54], sodium butyrate decreased the concentrations of all the four proinflammatory cytokines (IL-1*β*, IL-6, IL-8, and TNF-*α*) and APPs (CRP and Hp) in plasma, whereas sodium *β*-hydroxybutyrate only decreased the concentrations of IL-6, TNF-*α* and Hp in plasma. Thirdly, although both sodium butyrate and sodium *β*-hydroxybutyrate decreased the immune cells in blood and the SCC in milk, the effect of sodium butyrate was greater than sodium *β*-hydroxybutyrate. Finally, sodium butyrate decreased the expression of PRRs and proinflammatory cytokines in the mammary gland more effectively than sodium *β*-hydroxybutyrate.

## 4. Conclusions

In summary, although HC diet increased milk yield and decreased ketone body levels in the blood, it resulted in adverse effects on milk quality and the health of dairy cows. The adverse effects included a decrease in milk fat content and rumen pH as well as an increase in bacterial cell wall components (LPS, PGN, and LTA) in rumen fluid and plasma, proinflammatory cytokines and APPs in plasma, immune cells in blood, SCC in milk, and the gene expression of PRRs and proinflammatory cytokines in mammary cells. Sodium butyrate supplementation into HC diet not only increased milk yield and milk fat content, but also reduced the adverse effects caused by HC diet. Sodium *β*-hydroxybutyrate supplementation also increased milk yield and milk fat content and partially reduced the adverse effects caused by HC diet. Sodium butyrate was more effective than sodium *β*-hydroxybutyrate in mitigating the adverse effects of HC diet, because sodium butyrate more effectively decreased the concentrations of bacterial cell wall components (LPS, PGN, and LTA) in rumen fluid and plasma than sodium *β*-hydroxybutyrate. The work suggests that sodium butyrate and sodium *β*-hydroxybutyrate might be promising feed additives for HC diet of ruminants.

## 5. Materials and Methods

### 5.1. Ethics Statement

Both the feeding trial of dairy cows and the experimental sample collection were approved by Animal Ethics Committee of Southwest University (Number: 3167130267; Date: 19 January 2016), and all experimental procedures and the care of the animals were in strict accordance with the Guidelines on Ethical Treatment of Experimental Animals (2006, No. 398) issued by the Ministry of Science and Technology of China.

### 5.2. Chemicals and Reagents

Sodium butyrate was purchased from Dongying Dego Biotechnology, Co., Ltd. (http://jinan0108850.11467.com/ (accessed on 31 October 2019, Jinan, China)). Sodium *β*-hydroxybutyrate was purchased from Shanghai Kangxin Chemical Co., Ltd. (http://www.shkangxin.com.cn/ (accessed on 31 October 2019, Shanghai, China)). Analytical grade reagents were purchased from Chongqing Chuandong Chemical Co., Ltd. (Chongqing, China) and Chengdu Kelong Chemical Co., Ltd. (Chengdu, China).

### 5.3. Experimental Design, Dairy Cows, and Diets

This experiment was a completely randomized design trial with 20 replicates per group. Each dairy cow was an experimental unit, and all dairy cows were fed under the same condition before the start of the study. Dairy cows were randomized using a computer based random order generator. Eighty healthy Chinese Holstein cows (2 or 3 parity) at 96 ± 13 d (mean ± standard deviation) in milk, with an average milk yield of 33.75 ± 5.22 kg/d (mean ± standard deviation), were selected and randomly divided into 4 groups (n = 20 per group). The 4 groups of cows were administrated one of the following diets: a low-concentrate (LC) diet (concentrate:forage = 4:6), named the “LC” group; a HC diet (concentrate:forage = 6:4), named the “HC” group; the HC diet supplemented with 1% sodium butyrate, named the “HCSB” group; and the HC diet supplemented with 1% sodium *β*-hydroxybutyrate, named the “HCHB” group. Both sodium butyrate and *β*-hydroxybutyrate accounted for 1% of the diet DM. The 4 groups of dairy cows were selected from the same farm and group housed on the farm, without subjecting to transport stress and the change of environmental conditions. The ingredients and nutrient compositions of all the experimental diets are listed in Table 5. Collected feed samples were dried at 6 °C in an oven with forced air. Next, the nutrient compositions were analyzed according to the methods described by Zhang [56]. Only animal keepers were aware of the diets fed to each group of dairy cows, because the diets were freshly made and fed as total mixed ration (TMR) each time of feeding by the keepers. The cows had free access to TMR and water. The feeding trial lasted for a total of 7 weeks, with a 2-week adaptation period and a 5-week measurement period. All cows were milked at 8:00, 14:00 and 20:00 using an automatic milking system (GEA Farm Technologies, Dusseldorf, Germany). The research team, animal keepers, and veterinary staff monitored the dairy cows daily for their health conditions.

### 5.4. Sample Collection

The feed samples in each group were collected at the start of the experiment and stored at −20 °C for analyzing nutrient components of the diets. The milk samples were collected three times daily at 8:00, 14:00 and 20:00 and were mixed with a ratio of 4:3:3 on the day of collection. The mixed milk samples were immediately detected for milk components. The rumen fluid samples were collected 3 to 4 h after the morning feeding on the last two days of the feeding experiment using an oral stomach tube equipped with a 200 mL pyrogen-free syringe, as described by previous studies [1,2,38,57]. About 200 mL rumen fluid was filtered with a four-layers gauze. The collected filtrate was divided into two portions. One portion was immediately used to measure pH by a portable pH meter (Rex PHS-3E, Shanghai INESA Scientific Instrument Co., Ltd., Shanghai, China). Another portion (50 mL) was transferred into pyrogen-free centrifuge tubes and stored at −8 °C for detecting the bacterial cell wall components. Blood was obtained from the coccygeal vein before the morning feeding on the last two days and collected into two sets of pyrogen-free blood collection tubes (5 mL). One set (purple tubes) contained EDTA for measuring blood leucocyte indexes. The other one (green tubes) contained sodium heparin and was centrifuged at 3000× *g* for 15 min at 4 °C for plasma isolation. The isolated plasma was stored at −80 °C and used to detect the concentrations of bacterial cell wall components, proinflammatory cytokines, and APPs. If a dairy cow suffered from acute mastitis and other diseases, it was excluded from sampling.

### 5.5. Production Performance Measurement

The dairy cows were fed individually using Calan gates at feeding time. The TMR provided in the morning, noon, and evening as well as daily orts collected were used to calculate daily feed intake of each cows. The DMI was calculated by multiplying the daily feed intake with dry matter content of the TMR. The milk yield was measured in the morning, noon, and evening to obtain daily milk yield of each cow. The milk components (milk fat, milk protein, lactose, and solid-non-fat) were measured using a milk component analyzer (Speedy Lab, Poncarale, Italy). Thereafter, these data were used for calculation of milk synthesis efficiency and 4% FCM synthesis efficiency, as described by Zhou et al. [38].

### 5.6. Measurement of Bacterial Cell Wall Components

Ten samples in each group were randomly selected for measurement of bacterial cell wall components (n = 10 per group). The LPS concentrations in rumen fluid were measured by the End-point Chromogenic Lyophilized Tachypleus Amebocyte Lysate Kit (Xiamen Bioendo Technology Co., Ltd., Xiamen, China), with a detective range of 0.1–1 EU/mL. The plasma LPS concentrations were measured using the more sensitive End-point Chromogenic Lyophilized Tachypleus Amebocyte Lysate Kit (Xiamen Bioendo Technology Co., Ltd., Xiamen, China), with a detective range of 0.01–0.1 EU/mL. The sample processing for rumen fluid and plasma was described in detail in our previous studies [1,2,38]. The PGN concentrations in rumen fluid and plasma were measured by the GPB Peptidoglycan Chromogenic Assay Kit (Autobio Diagnostics Co., Ltd., Zhengzhou, China), a patent product from Autobio (patent number: CN201610230254.1). The LTA concentrations in rumen fluid and plasma were measured using the LTA ELISA Kit (J&L Biological Industrial Co., Ltd., Shanghai, China), as described by previous studies [58,59]. The rumen fluid samples were centrifuged and diluted to the detective ranges using pyrogen-free water and depyrogenated glass tubes. All the operational procedures were carried out in strict accordance with the manufacturer’s instructions. The optical density (OD) values were read by a microplate reader (BioTek, Winooski, VT, USA).

### 5.7. Detection of Proinflammatory Cytokines and APPs

Ten samples in each group were randomly selected for detection of proinflammatory cytokines and APPs (n = 10 per group). The concentrations of four proinflammatory cytokines (IL-1*β*, IL-6, IL-8, and TNF-*α*) were measured using the bovine ELISA kits (Sinobest Biotechnology Co., Ltd., Shanghai, China). The concentrations of APPs (LBP, SAA, CRP, and Hp) were measured using the bovine ELISA kits (Sinobest Biotechnology Co., Ltd., Shanghai, China). All the assays were performed according to the manufacturer’s instructions.

### 5.8. Measurements of Blood Leucocyte Indexes and Milk SCC

Ten samples in each group were randomly selected for measurement of blood leucocyte indexes and milk SCC (n = 10 per group). After blood was collected, it was immediately sent to the Southwest University Animal Hospital to measure blood leucocytes by an automatic hematology analyzer (Mindray, Shenzhen, China). The fresh milk samples collected in the morning were immediately measured for SCC using a somatic cell counter (DeLaval, Tumba, Sweden).

### 5.9. Detection of Blood Glucose and Blood Ketone Bodies

Ten samples in each group were randomly selected for detection of blood glucose and blood ketone bodies (n = 10 per group). After blood sampling, the fresh blood samples were immediately detected for blood glucose using a Sannuo blood glucose meter (Sinocare Biosensing Co., Ltd., Changsha, China) and blood ketone bodies using a blood ketone body meter specific for dairy cows (Tianyuannongren Biotech Co., Ltd., Beijing, China). The test strips were placed in the blood ketone body meter. Next, a small amount of fresh blood sample was carefully absorbed using the test strips. The measurement results were read after 30 s.

### 5.10. Analysis of Quantitative Real-Time Polymerase Chain Reaction (qRT-PCR)

The milk somatic cells collected were used to isolate RNA for the qRT-PCR (n = 6 per group). The specific procedure for isolation of milk somatic cells has been described in detail in previous studies [51,60]. The total RNA was isolated using the FastPure Cell/Tissue Total RNA Isolation Kit (Vazyme, Nanjing, China), and its concentration and quality were detected by a microvolume spectrophotometer (Implen, Munich, Germany). The RNA concentrations of all samples were diluted to 100 ng/μL using RNase-free water. Then, 4 μL RNA solution containing 400 ng of total RNA was used to synthesize complementary DNA (cDNA) using the reverse transcription kit (HiScript III RT SuperMix for qPCR (+gDNA wiper), Vazyme, Nanjing, China). The reverse transcription was performed in a T100™ thermal cycler (Bio-Rad, Hercules, CA, USA) according to the following incubation programs. Firstly, the genomic DNA was removed by incubation at 42 °C for 2 min. Next, the reverse transcription responses were conducted by incubation at 37 °C for 15 min and 85 °C for 5 s. The obtained cDNA templates were amplified using the ChamQ Universal SYBR qPCR Master Mix (Vazyme, Nanjing, China) and gene-specific primers on an ABI QuantStudio6 Q6 Real-Time PCR system (Applied Biosystems, Foster City, CA, USA). The amplification procedures were as follows: 95 °C for 30 s, 40 cycles at 95 °C for 10 s, and 60 °C for 30 s. In addition, a melting curve analysis (at 95 °C for 15 s, 60 °C for 60 s, and 95 °C for 15 s) was performed at the end of the amplification program to ensure specific amplification. The specific primers (Table S1) were designed using NCBI primer-BLAST (https://www.ncbi.nlm.nih.gov/tools/primer-blast/index.cgi (accessed on 6 August 2020)) and were synthesized and purified by Beijing Liuhe Huada Gene Technology Co., Ltd. (Beijing, China). The relative mRNA expression data of target genes were normalized by the glyceraldehyde-3-phosphate dehydrogenase (*GAPDH*) gene and calculated using 2^−ΔΔCT^ method. Six samples in each group were randomly selected for the qRT-PCR, and each cDNA sample was amplified three times.

### 5.11. Statistical Analysis

All the trial data were analyzed using IBM SPSS (version 19.0) statistic software (SPSS Inc., Chicago, IL, USA). General linear model with repeated measures was used to assess the statistical differences of DMI, milk yield, milk synthesis efficiency, 4% FCM synthesis efficiency, milk components, and SCC. One-way factorial analysis of variance followed by Duncan’s multiple range test was carried out to assess the statistical differences. The statistical differences were considered as significant if probability values were less than 0.05 (*p* < 0.05).

## Figures and Tables

**Table 1:** Rumen pH and the concentrations of LPS, PGN and LTA in the rumen and plasma.

Item	Diet				*p*-Value
	LC	HC	HCSB	HCHB	
rumen pH value	6.70 ± 0.18 ^ab^	6.24 ± 0.17 ^c^	6.72 ± 0.27 ^a^	6.53 ± 0.16 ^b^	<0.01
rumen LPS (EU/mL)	25,565 ± 3433 ^c^	68,777 ± 5978 ^a^	55,173 ± 6135 ^b^	65,310 ± 7391 ^a^	<0.01
rumen PGN (μg/mL)	42.9 ± 3.5 ^b^	122.4 ± 17.6 ^a^	106.7 ± 14.4 ^a^	121.1 ± 50.7 ^a^	<0.01
rumen LTA (μg/mL)	2.42 ± 0.50 ^c^	4.16 ± 0.81 ^a^	3.10 ± 0.56 ^b^	4.03 ± 0.58 ^a^	<0.01
plasma LPS (EU/mL)	0.07 ± 0.01 ^c^	0.23 ± 0.04 ^a^	0.20 ± 0.02 ^b^	0.23 ± 0.04 ^a^	<0.01
plasma PGN (pg/mL)	173.8 ± 149.0 ^b^	485.6 ± 207.5 ^a^	200.1 ± 77.8 ^b^	423.6 ± 197.9 ^a^	<0.01
plasma LTA (ng/mL)	11.5 ± 1.1 ^c^	16.9 ± 2.7 ^a^	14.1 ± 0.9 ^b^	16.3 ± 2.81 ^a^	<0.01

LPS: lipopolysaccharide; PGN: peptidoglycan; LTA: lipoteichoic acid; EU: endotoxin unit; LC: low concentrate diet; HC: high concentrate diet; HCSB: HC supplemented with 1% sodium butyrate; HCHB: HC supplemented with 1% sodium *β*-hydroxybutyrate. The data are presented as means ± standard deviation. Means in the same row without a common superscript are significantly different (*p* < 0.05, n = 10), based on one-way analysis of variance with Duncan’s multiple range test.

**Table 2:** Concentrations of proinflammatory cytokines and acute phase proteins in plasma.

Item	Diet				*p*-Value
	LC	HC	HCSB	HCHB	
Proinflammatory cytokines					
IL-1*β* (pg/mL)	122.9 ± 11.7 ^c^	169.4 ± 14.9 ^a^	140.4 ± 14.3 ^b^	162.3 ± 6.8 ^a^	<0.01
IL-6 (pg/mL)	161.1 ± 23.6 ^b^	203.5 ± 24.0 ^a^	169.9 ± 14.1 ^b^	178.9 ± 27.3 ^b^	<0.01
IL-8 (pg/mL)	272.7 ± 53.7 ^c^	346.1 ± 15.7 ^a^	297.1 ± 25.4 ^bc^	321.8 ± 30.1 ^ab^	<0.01
TNF-*α* (pg/mL)	1313 ± 111 ^b^	1499 ± 66 ^a^	1346 ± 73 ^b^	1368 ± 118 ^b^	<0.01
Acute phase proteins (APPs)					
LBP (μg/mL)	143.0 ± 25.2 ^b^	240.8 ± 23.3 ^a^	227.4 ± 20.5 ^a^	234.2 ± 20.4 ^a^	<0.01
SAA (μg/mL)	12.3 ± 1.6 ^b^	14.8 ± 2.0 ^a^	13.1 ± 0.8 ^ab^	14.5 ± 1.9 ^a^	0.02
CRP (mg/L)	10.4 ± 1.1 ^c^	12.5 ± 1.3 ^a^	10.9 ± 0.8 ^bc^	11.9 ± 0.9 ^ab^	<0.01
Hp (μg/mL)	1152 ± 326 ^c^	1688 ± 169 ^a^	1315 ± 143 ^b^	1244 ± 91 ^b^	<0.01

IL-1*β*: interleukin-1*β*; IL-6: interleukin-6; IL-8: interleukin-8; TNF-*α*: tumor necrosis factor-*α*; LBP: lipopolysaccharide binding protein; SAA: serum amyloid A; CRP: C-reactive protein; Hp: haptoglobin; LC: low concentrate diet; HC: high concentrate diet; HCSB: HC supplemented with 1% sodium butyrate; HCHB: HC supplemented with 1% sodium *β*-hydroxybutyrate. The data are presented as means ± standard deviation. Means in the same row without a common superscript are significantly different (*p* < 0.05, n = 10), based on one-way analysis of variance with Duncan’s multiple range test.

**Table 3:** Blood leukocyte indexes.

Index	Unit	Diet				*p*-Value
		LC	HC	HCSB	HCHB	
Number of white blood cells	10^9^/L	17.5 ± 4.9 ^b^	27.3 ± 5.2 ^a^	17.2 ± 8.0 ^b^	15.7 ± 5.3 ^b^	<0.01
Number of lymphocytes	10^9^/L	9.9 ± 3.6 ^b^	15.7 ± 4.1 ^a^	8.2 ± 6.1 ^b^	7.3 ± 4.5 ^b^	<0.01
Number of monocytes	10^9^/L	0.88 ± 0.29 ^b^	1.64 ± 0.76 ^a^	0.97 ± 0.43 ^b^	1.04 ± 0.43 ^ab^	<0.01
Number of neutrophils	10^9^/L	6.7 ± 1.6 ^b^	9.9 ± 3.2 ^a^	7.8 ± 2.0 ^a^	7.3 ± 1.5 ^a^	0.01
Ratio of lymphocytes	%	55.2 ± 8.2 ^a^	57.5 ± 10.2 ^a^	42.8 ± 14.8 ^b^	43.3 ± 14.7 ^b^	0.01
Ratio of monocytes	%	5.3 ± 1.5 ^b^	6.1 ± 3.0 ^ab^	7.6 ± 2.3 ^a^	6.9 ± 1.8 ^ab^	0.13
Ratio of neutrophils	%	39.5 ± 7.1 ^ab^	36.4 ± 9.7 ^b^	49.6 ± 13.1 ^a^	49.8 ± 13.9 ^a^	0.02

LC: low concentrate diet; HC: high concentrate diet; HCSB: HC supplemented with 1% sodium butyrate; HCHB: HC supplemented with 1% sodium *β*-hydroxybutyrate. The data are presented as means ± standard deviation. Means in the same row without a common superscript are significantly different (*p* < 0.05, n = 10), based on one-way analysis of variance with Duncan’s multiple range test.

**Table 4:** The concentrations of glucose and ketone bodies in blood.

Item	Diet				*p*-Value
	LC	HC	HCSB	HCHB	
Blood glucose (mmol/L)	2.33 ± 0.41	2.52 ± 0.62	2.69 ± 0.50	2.74 ± 0.46	0.27
Blood ketone bodies (mmol/L)	0.71 ± 0.07 ^b^	0.62 ± 0.08 ^c^	0.71 ± 0.07 ^b^	0.79 ± 0.07 ^a^	<0.01
Blood ketone bodies (mg/dL)	7.42 ± 0.72 ^b^	6.66 ± 0.85 ^c^	7.48 ± 0.58 ^b^	8.37 ± 0.65 ^a^	<0.01

LC: low concentrate diet; HC: high concentrate diet; HCSB: HC supplemented with 1% sodium butyrate; HCHB: HC supplemented with 1% sodium *β*-hydroxybutyrate. The concentrations of ketone bodies in blood were measured as *β*-hydroxybutyrate concentrations in blood. The data are presented as means ± standard deviation. Means in the same row without a common superscript are significantly different (*p* < 0.05, n = 10), based on one-way analysis of variance with Duncan’s multiple range test.

**Table 5:** Ingredients and nutrient compositions of trial diets.

Item	Diet ^1^			
	LC	HC	HCSB	HCHB
Ingredient (% DM)				
*Leymus chinensis*	14.81	6.04	5.98	5.98
Corn silage	22.02	13.96	13.82	13.82
Alfalfa hay	12.77	10.97	10.86	10.86
Fresh distiller’s grains	5.87	3.64	3.60	3.60
Sugar beet meal	4.36	3.88	3.84	3.84
Cottonseed	0.00	1.75	1.73	1.73
Corn	21.80	32.36	32.04	32.04
Wheat bran	1.53	3.46	3.43	3.43
Palm oil	1.09	1.56	1.54	1.54
Soybean meal	5.43	7.77	7.69	7.69
Cottonseed meal	2.77	3.97	3.93	3.93
Distillers dried grain with solubles (DDGS)	3.93	5.62	5.56	5.56
Calcium hydrogen phosphate	1.10	1.58	1.56	1.56
Calcium carbonate (light powder)	0.66	0.95	0.94	0.94
Magnesium oxide	0.22	0.32	0.32	0.32
Salt	0.44	0.48	0.48	0.48
Vitamin and mineral premix ^2^	0.88	1.26	1.25	1.25
Urea premix	0.22	0.32	0.32	0.32
Yeast premix	0.09	0.13	0.13	0.13
Sodium butyrate	0.00	0.00	1.00	0.00
Sodium *β*-hydroxybutyrate	0.00	0.00	0.00	1.00
Concentrate:forage	4:6	6:4	6:4	6:4
Nutrient composition ^3^				
Net energy for lactation (NEL, MJ/Kg)	6.18	6.56	6.49	6.49
Crude protein (% DM)	15.4	16.4	16.3	16.2
Ether extract (% DM)	5.2	6.1	5.4	5.8
Neutral detergent fiber (% DM)	49.3	43.8	44.4	43.7
Acid detergent fiber (% DM)	22.4	18.9	18.8	18.3
Calcium (% DM)	1.02	1.03	1.04	1.06
Total phosphorus (% DM)	0.52	0.52	0.54	0.52

^1^ LC: low concentrate diet (concentrate:forage = 4:6); HC: high concentrate diet (concentrate:forage = 6:4); HCSB: HC diet supplemented with 1% sodium butyrate; HCSB: HC diet supplemented with 1% sodium β-hydroxybutyrate. ^2^ The vitamin and mineral premix contained the following: vitamin A (540 × 10^3^ IU/kg), vitamin D3 (135 × 10^3^ IU/kg), vitamin E (2.70 × 10^3^ mg/kg), biotin (5.4 mg/kg), Mn (1.35 × 10^3^ mg/kg), Cu (1.35 × 10^3^ mg/kg), Zn (6.75 × 10^3^ mg/kg), I (90 mg/kg), Se (35 mg/kg), and Co (20 mg/kg). ^3^ The net energy for lactation (NEL, MJ/Kg) was calculated values, and the remaining nutrient compositions (% DM) were measured values.

## Data Availability

Not applicable.

## Supplementary Materials

The following are available online at https://www.mdpi.com/article/10.3390/toxins13050352/s1, Table S1: The primer sequences of target genes and the internal reference gene (GAPDH).
