# Supplementary material for: Sodium Butyrate More Effectively Mitigates the Negative Effects of High-Concentrate Diet in Dairy Cows than Sodium β-Hydroxybutyrate via Reducing Free Bacterial Cell Wall Components in Rumen Fluid and Plasma
Source: Toxins (Basel). 2021 May 14;13(5):352. doi: 10.3390/toxins13050352 (PMC8157208; doi:10.3390/toxins13050352)
Supplement: Supplementary file 1 [file toxins-13-00352-s001.zip › toxins-1213509-supplementary.pdf]

Supplementary Materials: Sodium Butyrate More Effectively Mitigates the Negative Effects of High-Concentrate Diet in Dairy Cows than Sodium *β*-Hydroxybutyrate via Reducing Free Bacterial Cell Wall Components in Rumen Fluid and Plasma

Yongjiang Wu, Yawang Sun, Ruiming Zhang, Tianle He, Ke Tian, Junhui Liu, Juncai Chen and Guozhong Dong

Table S1. The primer sequences of target genes and the internal reference gene (*GAPDH*).

Gene.	Primer Sequence (5′ to 3′)	Product Size/bp	NCBI Accession No.
IL-1β	F: AGTGCCTACGCACATGTCTTC<br>R: TGCGTCACACAGAAACTCGTC	114	NM_174093.1
IL-6	F: TGCTGGTCTTCTGGAGTATC<br>R: GTGGCTGGAGTGGTTATTAG	153	NM_173923.2
IL-8	F: ATGACTTCCAAGCTGGCTGTTG<br>R: TTGATAAATTTGGGGTGGAAAG	149	NM_173925.2
TNF-α	F: CCACGTTGTAGCCGACATC<br>R: CCCTGAAGAGGACCTGTGAG	155	XM_005223596.4
TLR4	F: AACGAAAGCAGAAAGCCACAG<br>R: GACCAGGGGCTGAAGTAACAA	146	NM_174198.6
PGLYRP2	F: CAGGGCATGAGCCAAGAAGA<br>R: GAGCCCACCACGAAACTGTA	299	XM_002688558.5
TLR2	F: TCTGCTCTTCAGGCAAAATTC<br>R: CCAGTGATTTGACCTCCACTCT	103	NM_174197.2
NOD1	F: TGATGACGAAGTGGCAGAGAG<br>R: CCCACGAGAAAACATCCCCT	242	NM_001256563.1
GAPDH	F: GGGTCATCATCTCTGCACCT<br>R: GGTCATAAGTCCCTCCACGA	176	NM_001034034.2

IL-1β: interleukin-1*β*; *IL-6*: interleukin-6; *IL-8*: interleukin-8; *TNF-α*: tumor necrosis factor-*α*; *TLR4*: Toll-like receptor 4; *PGLYRP2*: Peptidoglycan recognition protein 2; *TLR2*: Toll-like receptor 2; *NOD1*: nucleotide-binding oligomerization domain 1; *GAPDH*: glyceraldehyde-3-phosphate dehydrogenase.
